# Dr. Miller’s Paper on Fermentation

**Published:** 1886-03

**Authors:** 


### DR. MILLER'S PAPER ON FERMENTATION.

We hope that all of our reflective readers will carefully study the series of papers now in course of publication by Dr. Miller. To the physiologist they are of absorbing interest, and, so far as we know, the demonstrations which he presents are entirely unique and must attract attention in advanced medical circles. It should be a matter of pride to us that an American dentist presents such a series of elaborate and conclusive experiments, and acts as a teacher to all medical men. His researches are too technical and learned for every dentist readily to follow, and they demand careful study from even the most advanced. But we can all feel a just exultation in his fame as an experimenter and observer, for his studies reflect credit upon his profession. We happen to know that some of the foremost physiologists and pathologists of America are watching his progress with absorbing interest

